# Advances in Infant Cry Paralinguistic Classification—Methods, Implementation, and Applications: Systematic Review

**DOI:** 10.2196/69457

**Published:** 2025-04-29

**Authors:** Geofrey Owino, Bernard Shibwabo

**Affiliations:** 1 School of Computing and Engineering Sciences Strathmore University Nairobi Kenya

**Keywords:** infant cry classification, machine learning, audio feature extraction, pitch analysis, hybrid models, denoising techniques, federated learning, real-time analysis, pediatric healthcare, signal processing, data confidentiality

## Abstract

**Background:**

Effective communication is essential for human interaction; yet, infants can only express their needs through various types of suggestive cries. Traditional approaches of interpreting infant cries are often subjective, inconsistent, and slow, leaving gaps in timely, precise caregiving responses. A precise interpretation of infant cries can potentially provide valuable insights into the infant’s health, needs, and well-being, enabling prompt medical or caregiving actions.

**Objective:**

This study seeks to systematically review the advancements in methods, coverage, deployment schemes, and applications of infant cry classification over the last 24 years. The review focuses on the different infant cry classification techniques, feature extraction methods, and practical applications. Furthermore, we aimed to identify recent trends and directions in the field of infant cry signal processing to address both academic and practical needs.

**Methods:**

A systematic literature review was conducted using 9 electronic databases: Cochrane Database of Systematic Reviews, JSTOR, Web of Science Core Collection, Scopus, PubMed, ACM, MEDLINE, IEEE Xplore, and Google Scholar. A total of 5904 search results were initially retrieved, with 126 studies meeting the eligibility criteria after screening by 2 independent reviewers. The methodological quality of these studies was assessed using the Cochrane risk of bias tool (version 2; RoB2), with 92% (n=116) of the studies indicating a low risk of bias and 8% (n=10) of the studies showing some concerns regarding bias. The overall quality assessment was performed using TRIPOD (Transparent Reporting of a Multivariable Prediction Model for Individual Prognosis or Diagnosis) guidelines. The data analysis was conducted using R (version 3.64; R Foundation).

**Results:**

Notable advancements in infant cry classification methods were realized, particularly from 2019 onward, using machine learning, deep learning, and hybrid approaches. Common audio features included Mel-frequency cepstral coefficients, spectrograms, pitch, duration, intensity, formants, 0-crossing rate, and chroma. Deployment methods included mobile apps and web-based platforms for real-time analysis, with 90% (n=113) of the remaining models remaining undeployed to real-world applications. Denoising techniques and federated learning were limitedly used to enhance model robustness and ensure data confidentiality from 5% (n=6) of the studies. Some of the practical applications spanned health care monitoring, diagnostics, and caregiver support.

**Conclusions:**

The evolution of infant cry classification methods has progressed from traditional classical statistical methods to machine learning models but with minimal considerations of data privacy, confidentiality, and ultimate deployment to practical use. Further research is thus proposed to develop standardized foundational audio multimodal approaches, incorporating a broader range of audio features and ensuring data confidentiality through methods such as federated learning. Furthermore, a preliminary layer is proposed for denoising the cry signal before the feature extraction stage. These improvements will enhance the accuracy, generalizability, and practical applicability of infant cry classification models in diverse health care settings.

## Introduction

Infants can only express their needs through cries as a way of communication before developing proper linguistic ontological organs as they grow. During these early stages, their needs are crucial and must be promptly attended to, as they are vulnerable to neglecting basic needs such as sleep, diaper changes, medical care, and general attention. Misunderstanding their needs can exacerbate problems, leading to further complications and health-related issues as they continue to cry if their needs are unmet [1]. Understanding and addressing the needs and health conditions of newborns are therefore paramount, given that infants cry an average of 25 minutes per day in their first week, increasing to nearly 2.25 hours daily by 6 weeks [1,2]. Infant cry analysis is crucial for identifying medical conditions such as neurological disorders, respiratory issues, and early signs of sepsis [3], as well as aiding in infant care and bonding [4]. Historically, the study of infant cries began in the 1960s, with Wasz-Höckert et al [5] categorizing distinct types of cries related to pain, hunger, birth, and pleasure through auditory analysis by trained nurses.

Infant cries usually serve as the first form of early verbal communication, conveying both physiological and emotional needs to parents and caregivers such as nannies [6]. Different infants’ cries tend to have associative distinctive acoustic signals that can be characterized by variations in pitch, duration, and intensity, providing vital information about an infant’s well-being [7]. Timely and precise response to these cues is linked to the infant’s continuous emotional development, according to the study by Caulfield [8].

In most instances, traditional methods of infant cry classification rely on manual subjective observations and rudimentary acoustic features, which inadequately capture the rich spectrum of cries and their underlying causes with less accuracy and reliability [9]. The field of parenting and caregiving has been phenomenally enriched with the adoption of machine learning and neural networking that can process complex audio datasets efficiently for so many diversified pattern identifications in infants’ cries. These technologies have not only enhanced the accuracy of infant cry paralinguistics analysis and discriminations but also allowed real-time analysis, which is crucial in health care settings anytime and anywhere. In comparison, this could also substantially accelerate the achievement of sustainable universal health coverage for infants’ well-being and growth when properly adopted and implemented.

Early research on infant cry classification, such as the use and adoption of machine learning algorithms by Mukhopadhyay et al [10], demonstrated a substantial significant improvement in accuracy over human listeners. However, most traditional observational methods, such as the Neonatal Infant Pain Scale [11] and the FLACC (Face, Legs, Activity, Cry, Consolability) scale [12], were subjective and significantly varied among different observers. This variability, therefore, highlighted the need for objective tools and technologies to measure and categorize infant cries.

The integration of advanced neural network architectures, such as convolutional neural networks and recurrent neural network models, has shown promise in capturing complex patterns in audio data for spatial-temporal components of the signals. Most recent advancements in signal processing tend to leverage the power of hybrid models, such as convolutional recurrent neural networks and ensemble modeling, which potentially capture both spatial and temporal information within cry signals [13]. Current research has limitedly also explored state-of-the-art federated learning for privacy-preserving analysis and the use of denoising techniques to enhance model robustness for signal data points.

The evolution of infant cry analysis reflects a continuous effort to move from subjective assessments to more objective, quantitative, and technologically advanced approaches. This progression aims to improve the early detection of health issues and facilitate timely interventions, ultimately enhancing the quality of infant care. This study, therefore, seeks to systematically review the advances in infant cry classification, encompassing a broad spectrum of methodologies, deployment strategies, and applications. Given the diversity of approaches, spanning from traditional observational techniques to modern machine learning and deep neural networks, the following relevant key research questions were considered: How do the reviewed key models generalize across diverse infant populations given the variability in cry characteristics?

This study discusses various methods, such as auditory analysis, signal processing, and statistical approaches, with ultimate deployment in real-world scenarios. It provides a comprehensive overview of how these empirical methodologies have evolved and their current applications in enhancing the accuracy and effectiveness of infant cry classification anytime and anywhere.

## Methods

### Goal and Review Questions

The main goal of this study was to systematically review and synthesize studies that focused on the classification and interpretation of infant cries. This includes an in-depth examination of the methodologies, deployment strategies, and the ultimate practical applications of these techniques.

Furthermore, this study aimed to identify recent trends, coverage, and directions in the field of audio classification to highlight opportunities for future research from both academic and practical perspectives. Based on the established guidelines for conducting systematic literature reviews by 2 studies [14,15] and for reporting findings by 2 other studies [16,17], the selection and review process was thoroughly developed and documented. Based on this goal, the following review questions (as illustrated in Table 1) were formulated and adopted:

**Table 1:** RQs^a^.

Category and RQ		RQ	Focus
**General RQs**			
	RQ 1.1	Contributions of the studies	Methods, models, tools, or applications for infant cry classification
	RQ 1.2	Research methods used	Empirical, theoretical, or hybrid approaches
	RQ 1.3	Geographical distribution of studies	Regional trends, dominant research locations, and gaps in global representation
**Topic-specific RQs**			
	RQ 2.1	Methods for cry classification	Statistical models, traditional techniques, or machine learning
	RQ 2.2	Feature extraction methods	MFCC^b^, spectrogram, or other audio features
	RQ 2.3	Types of datasets used	Primary (collected) or secondary (public repositories)
	RQ 2.4	Accuracy and precision	Performance comparison of methods or models
	RQ 2.5	Denoising techniques	First-layer noise reduction methods
	RQ 2.6	Data confidentiality and privacy	Federated learning or other privacy measures
	RQ 2.7	Model deployment	Practical use in real-world applications
	RQ 2.8	Applications of findings	Hospitals, home care, or other settings
	RQ 2.9	Limitations and challenges	Data quality, model robustness, and generalizability

^a^RQ: review question.

^b^MFCC: Mel-frequency cepstral coefficient.

### Search Strategy

To ensure transparency and completeness in our systematic review, we followed the PRISMA (Preferred Reporting Items for Systematic Reviews and Meta-Analyses) guidelines as outlined by 2 studies (the PRISMA checklist provided in Multimedia Appendix 1) [16,17]. Our search spanned 7 databases, covering literature from their inception up to 2024, to identify relevant articles on the methodologies, deployment strategies, and practical applications in infant cry classification. This broad time frame ensures the inclusion of both foundational and recent advancements, allowing for a comprehensive understanding of research trends [9]. Previous systematic reviews have similarly adopted this approach to track methodological evolution and identify gaps in infant cry analysis [1]. These databases comprised the Cochrane Database of Systematic Reviews, Web of Science Core Collection, Scopus, PubMed, ACM, IEEE Xplore, and Google Scholar. Our search strings, structured according to the PICO (Problem, Intervention, Comparison, and Outcome) framework by Petersen et al [18] (Multimedia Appendix 2), were designed to address the overarching question: “Can advanced methodologies and models enhance the classification and interpretation of infant cries?” We tailored the PICO structure as follows:

1. Problem: We searched for studies focusing on infant cry classification and its various aspects, encompassing terms such as “infant cry” OR “infant crying” OR “baby cry” and “infant vocalizations.”
2. Intervention: Our search targeted studies exploring methodologies, techniques, and approaches used for infant cry classification, including terms such as “machine learning” OR “neural networks” OR “signal processing” OR “auditory analysis” and “spectrogram.”
3. Comparison: Due to the specificity of our research objectives, direct comparison studies were not applicable in this context.
4. Outcome: We sought outcomes related to model accuracy, deployment, and practical applications of infant cry classification methodologies, incorporating terms such as “accuracy” OR “deployment” OR “application” and “real-time analysis.”

By using “AND” and “OR” operators, we formulated a general search phrase: (“infant cry” OR “infant crying” OR “baby cry” OR “infant vocalizations”) AND (“machine learning” OR “neural networks” OR “signal processing” OR “auditory analysis” OR “spectrogram”) AND (“accuracy” OR “deployment” OR “application” OR “real-time analysis”). The variations in these search phrases were adapted accordingly depending on the specifications of different databases that were taken into consideration (Multimedia Appendix 3).

### Eligibility Criteria

#### Overview

The eligibility criteria for inclusion were established to comprehensively evaluate studies that focused on the classification and interpretation of infant cries, considering a wide range of methodologies and relevant research. The criteria were precisely crafted to ensure the inclusion of studies that aligned with the objectives of this review. The studies were also evaluated to check if they were addressing the diverse needs and challenges in the methodologies, deployment schemes, and practical applications of infant cry classification in different domains of interest.

#### Initial Screening

During this preliminary phase, articles that were identified through database searches and manual exploration underwent early screening based on titles and abstracts. The aim was to ensure that the screened studies were aligning with the study’s goal of systematically reviewing studies on infant cry classification. Emphasis was placed on filtering out irrelevant studies and identifying those contributing to the examination of methodologies, deployment strategies, practical applications, recent trends, and future research opportunities. Adherence to established guidelines ensured comprehensive documentation of the selection process, facilitating the formulation of research questions and ultimate findings.

### Inclusion and Exclusion

#### Overview

Out of 5904 search results, a total of 126 studies were included for review. Studies that were included must have fulfilled the following criteria: (1) studies that focused on methods for infant cry classification, (2) studies that detailed the deployment strategies of these methods, and (3) studies that clearly defined practical applications of cry classification techniques.

To ensure precise and robust findings, this study used exclusion criteria guided by specific thematic considerations. (1) Irrelevant topics: Literature investigating other aspects of infant behavior not related to cry classification. (2) Incomplete focus: Studies focused solely on other signal processing tasks, such as noise reduction without subsequent classification. (3) Lack of technological interventions: Literature that did not apply technological interventions, such as those focusing purely on theoretical aspects without practical deployment. (4) Insufficient application: Literature that only described features of infant cries without using them in classification models. (5) Limited scope: Studies that solely focused on the intelligibility of infant cries without addressing their classification. (6) Non-English publications: Studies that were not available in English due to language proficiency constraints of the review team. The 10 additional papers were identified using citation tracking and backward snowballing techniques, ensuring that they served as anchor papers closely aligned with this study’s focus on infant cry classification, feature extraction methods, and deployment strategies.

A 2-stage screening process was used to ensure thoroughness and reduce the bias arising. Two reviewers independently screened all titles and the corresponding abstracts in the first step to assess relevance based on the inclusion criteria. Afterward, the papers were advanced to the next stage if and when at least one reviewer deemed them relevant. In cases where consensus at the title and abstract level could not be achieved between the 2 reviewers, an adjudication by a third person was sought on a consultation basis and on the purpose. During the second stage, 2 independent reviewers performed a full-text blind review. Consensus was obtained through deliberation between reviewers when required. Consensus meetings were held to resolve any disagreements and uncertainties. The objectivity of the criteria was assessed either prereview on a test set by measuring agreement or postreview.

#### Data Synthesis and Comprehensive Analytical Approach

In alignment with the review questions, the following information points were recorded and analyzed: (1) year of publication, (2) contribution of each study, (3) categorization of each study per the relevant research method type, (4) classification techniques and methods used in each study, (5) nature of feature extraction methods used, (6) deployment strategies discussed, (7) practical applications and settings where the techniques were applied (eg, hospitals or home-based care), (8) reported accuracy levels of the classification methods, and (9) data sources used in the studies. Tables and graphs were developed to summarize these information points, providing a comprehensive overview of the advances in infant cry classification methods, deployment strategies, and practical applications. The oldest paper in our analysis was from 2003, with a trend of increasingly more publications Table 2, which speaks to how this is an emerging area of research and interest.

**Table 2:** Distribution of study designs and deployment strategies.

Study design and deployment strategy	Values, %
Not deployed	109 (86.5)
Experimental study design	103 (81.7)
Primary dataset used	85 (66.7)
Secondary dataset used	46 (36.5)
Denoising performed	31 (24.6)
Descriptive study design	20 (15.1)
Correlational study design	11 (8.7)
Confidentiality compliance	10 (7.9)
Quasi experimental or causal-comparative study design	9 (7.1)
Software	6 (4.0)
Android	4 (2.4)
IoT^a^	3 (1.6)
Web	2 (0.8)

^a^IoT: Internet of Things.

#### Quality Assessment

The objective of this quality assessment was to evaluate the significance and validity of each selected document in the context of advances in infant cry classification. As much as the quality rating did not significantly influence the selection of the primary investigations, the assessment aimed to reflect the overall validity of the chosen studies. The TRIPOD (Transparent Reporting of a Multivariable Prediction Model for Individual Prognosis or Diagnosis) standard [19] was used as a measure of conventional quality standards. Each paper was scored based on adherence to the TRIPOD checklist, with binary scores assigned as either 1 (compliant) or 0 (noncompliant). Moreover, all the reviewed papers were additionally required to achieve a score higher than 50%. For a detailed checklist of the TRIPOD assessment, see Multimedia Appendix 4.

The TRIPOD checklist was used to evaluate the reporting clarity and comprehensiveness across all the studies that were considered and selected. Each study was evaluated by the dint of key TRIPOD components, with a maximum score of 5 points. All studies achieved the highest score, reflecting ample adherence to the TRIPOD guidelines. The high degree of compliance ensured transparency and thoroughness in reporting, thereby facilitating the reproducibility of the final study results.

This study relied on the Cochrane Risk of Bias 2 (RoB2) tool to assess the risk of bias (RoB) in the selected studies. Given that the main participants of the reviewed studies were infants with varied audio cry characteristics, the RoB2 tool was deemed appropriate for evaluating domain-specific and overall risks of bias. The RoB assessment covered the following five key domains: (1) bias arising from the randomization process, (2) bias due to deviations from intended interventions, (3) bias arising from missing outcome data, (4) bias in the measurement of outcomes, and (5) bias in the selection of reported results. The results of the RoB assessments were summarized in Table 3, and detailed individual RoB ratings were made available in Multimedia Appendix 5.

**Table 3:** RoB^a^ assessment.

RoB	Low, n	Some concerns, n	No information, n
Randomization process	121	1	4
Deviations of interventions	125	1	0
Missing outcome data	31	95	0
Measurement of outcome	124	2	0
Selection of reported result	122	4	0
RoB overall	116	10	0

^a^RoB: risk of bias.

The assessment of the randomization process bias indicated that most studies, 96% (n=121), were considered to have a low RoB, suggesting minimal influence from human choices or external factors on outcomes. A small portion, 1% (n=1) [20], raised some concerns, while 3% (n=4) lacked sufficient information to assess bias accurately. These findings imply that, overall, randomization in most studies was robust, supporting the credibility of their results.

The assessment of deviation from intended interventions bias showed that 99% (n=125) of studies had a low RoB, indicating a strong alignment between intended and actual interventions. Only 1% (n=1) [21] of studies presented some concerns, suggesting that, overall, most studies maintained consistency in their intervention protocols, minimizing the RoB in this area.

The assessment of missing outcome data bias revealed a significant area of concern, with 75% (n=95) of studies flagged for potential bias due to incomplete or missing outcome data. Only 25% (n=31) of studies were considered low risk in this domain, suggesting that missing data may introduce biases that could affect the reliability of the study outcomes and their interpretation.

The assessment of measurement of outcomes bias indicated that 97% (n=122) of studies were at low risk, demonstrating the use of robust measurement techniques across most studies. Only 3% (n=4) showed some concerns, suggesting that outcome assessments were generally reliable and unlikely to introduce significant bias.

The assessment of selection of reported results bias showed that 97% (n=122) of studies were at low risk, indicating that most studies reported results comprehensively without selective bias. Only 3% (n=4) raised minor concerns, suggesting minimal issues with incomplete reporting across the studies.

The overall assessment of RoB indicated that 92% (n=116) of the studies posed a low risk, while 8% (n=10) showed some concerns. These concerns were mainly related to handling and reporting missing outcome data, suggesting that most studies were methodologically sound, with only a few displaying minor issues that could impact data reliability.

**Figure 1:**
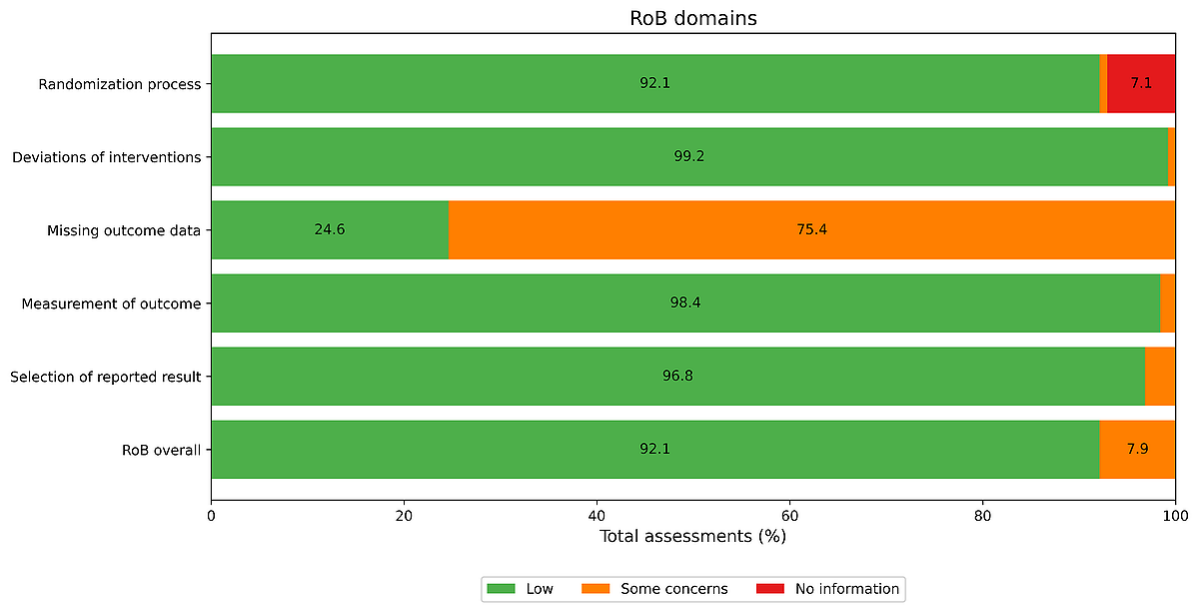
Visual representation of the distribution of RoB. RoB: risk of bias.

The RoB2 assessment affirmed the overall reliability of the included studies, with low risk across most domains that were of interest. The TRIPOD assessment, however, confirmed that all studies met the reporting standards necessary for transparency and reproducibility, achieving the maximum score. The Cochrane RoB2 assessment supported the robustness of the reviewed studies, identifying consistently low RoB across most domains. However, missing outcome data was the primary area of concern in a subset of different studies. These findings reinforced the methodological soundness of the included research, contributing valuable insights to the field of infant cry classification as a whole. Figure 1 provides a visual representation of the distribution of risk across all domains of interest, highlighting the minimal overall RoB.

## Results

### Overview

**Figure 2:**
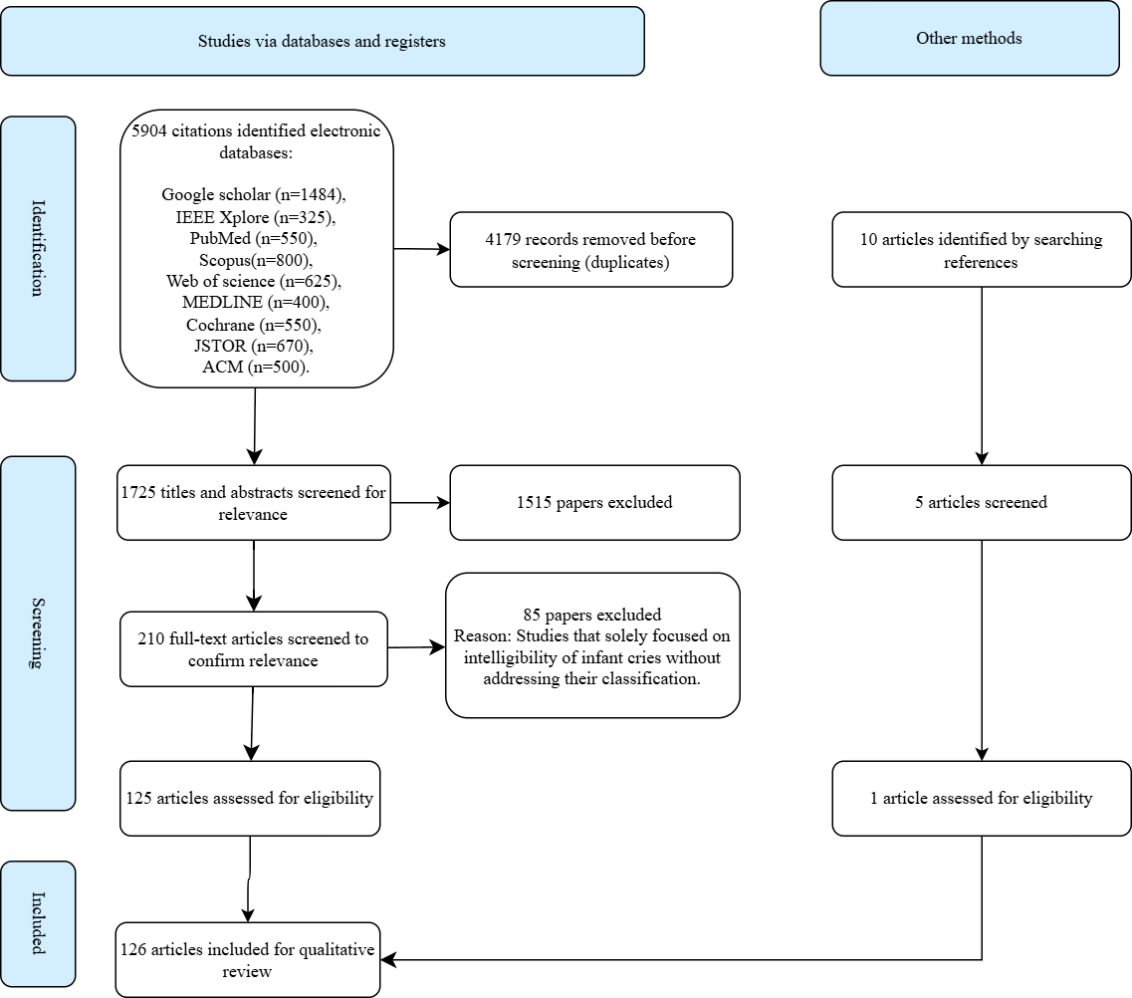
PRISMA flowchart. PRISMA: Preferred Reporting Items for Systematic Reviews and Meta-Analyses.

The findings in this section have been structured according to the classification of studies reviewed and the technical considerations for contextualizing infant cry classification. The selection process was seamlessly done, as presented in Figure 2.

### Feature and Method Use Across Studies

**Figure 3:**
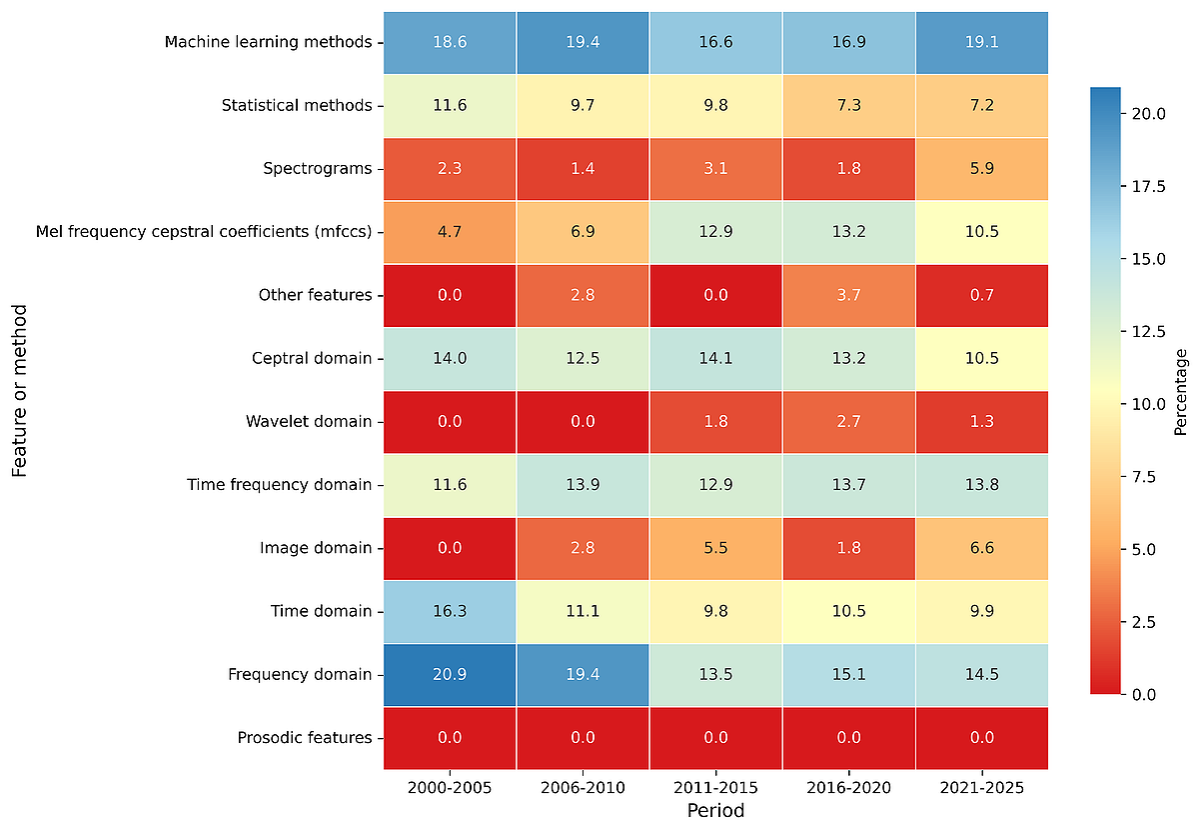
Proportion of features and methods used over time.

**Figure 4:**
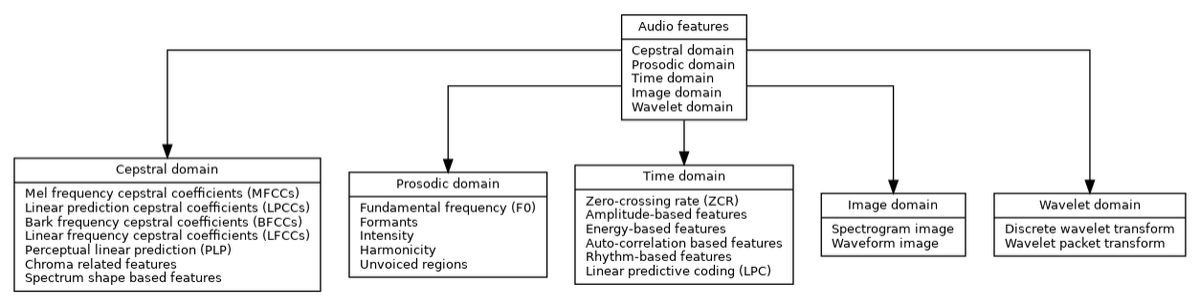
Audio features relevant in classification. BFCC: Bark frequency cepstral coefficient; F0: fundamental frequency; LFCC: linear frequency cepstral coefficients; LPC: linear predictive coding; LPCC: linear prediction cepstral coefficients; MFCC: Mel-frequency cepstral coefficient; PLP: perceptual linear predictive coefficient; ZCR: 0-crossing rate.

The analysis of the reviewed studies revealed that there was a varied distribution of features and methods that were adopted for infant cry classification. According to the results from Figure 3, machine learning methods were the most applied techniques, used in 91.3% (n=115) of the studies that were reviewed. This reflects the increasing trend toward using machine learning models for complex data tasks in infant cry classification. Figure 4 shows the various features used by authors in classification.

Frequency domain features appeared in 79.4% (n=100) of the studies, citing their significance in signal processing tasks related to cry analysis. Other prominent methods included the time frequency domain 69% (n=87), cepstral domain 65.9% (n=83), and Mel-frequency cepstral coefficients (MFCCs) 57.9% (n=73), which are well-established in speech and audio signal processing. These methods highlighted the relevance of time-frequency representations and feature extraction in understanding infant cry patterns.

A relatively lower adoption was noted in image domain techniques 19.8% (n=25) and spectrograms 15.9% (n=20), which are visual representations of sound but are gaining traction in more recent studies. The wavelet domain and other features both constituted 8.7% (n=11) of the studies and were among the least used, while prosodic features were realized not to have been applied in any of the reviewed studies. The other features category (n=11, 8.7%) included nonlinear dynamical features such as Lyapunov exponents, correlation dimension, and entropy measures, as well as physiologically inspired features such as Bark frequency cepstral coefficients, Gammatone cepstral coefficients, and perceptual features such as perceptual linear predictive coefficients and harmonic-to-noise ratio. These findings suggest a strong preference for machine learning and signal processing techniques, particularly those that are rooted in frequency and time-frequency analysis domain, as core methodologies in infant cry classification research.

### Temporal Analysis of Feature and Method Use

The evolution of feature and method use across the reviewed studies was assessed over different 5-year time stamp periods, as shown in Figure 3. This analysis shows the findings of trends in the adoption of various techniques for infant cry classification between the years 2000 and 2024. From the study results, machine learning methods have shown a steady increase over time, starting at 18.6% (n=23) in the 2000-2005 period and rising to 19.1% (n=24) in 2021-2024. This reflects the growing reliance on machine learning models in cry classification tasks, particularly in recent years. Frequency domain features consistently remained a major component across all periods, with a peak use of 20.9% (n=26) between the years 2000 and 2005, though their application saw a slight fall to 14.5% (n=18) in the most recent period (2021-2024). MFCCs exhibited a notable rise in use from 4.7% (n=6) in the early 2000s to 13.2% (n=17) by 2016-2020, thereby affirming their importance in speech and cry signal processing–related tasks. Spectrograms and image domain features, although less prominent overall, demonstrated growth in adoption over time, particularly in the 2021-2025 period, where the use of spectrograms significantly increased to 5.9% (n=7), and the image domain rose to 6.6% (n=8). Statistical methods, however, showed a decline from 11.6% (n=15) in the 2000-2005 period to 7.2% (n=9) in 2021-2025, indicating a shift toward more sophisticated machine learning approaches over traditional statistical models. Time-frequency domain and cepstral domain methods maintained a relatively consistent usage across all periods, suggesting their continued potential relevance in the analysis of infant cry signals. Some methods, such as wavelet domain and other features, remained minimally applied, reflecting limited exploration in these areas compared to more dominant techniques such as machine learning. This temporal trend analysis highlights the increasing sophistication and diversification of methodologies applied in infant cry classification research over time. The growing use of machine learning and feature extraction techniques, such as MFCCs and spectrograms, indicates a shift toward more complex, data-driven approaches in the field.

### Regional Distribution of Published Papers

The geographical distribution of published papers in the field of infant cry classification was one of the areas of interest and is summarized in Table 3. The data reveal a significant concentration of research efforts in specific regions, with Asia contributing to the highest proportion of papers, accounting for 54% (n=68) of the total. Most of these papers, 66.7% (n=45), were both primary and secondary research, indicating a strong engagement in both foundational and application-based studies in the region. Europe came at the second most active region, publishing 27.4% (n=36) of the total papers in this line of infant cries classification, with a notable portion of these being primary studies 30.3% (n=11). A further 16.7% (n=6) of European publications contributed to both primary and secondary research, while 26.3% (n=9) were secondary studies. In contrast, North America, including Canada and the United States, showed more limited contributions. The United States accounted for 9.7% (n=12) of the total papers, with the majority being primary studies 13.6% (n=2), and 5.3% (n=1) focused on secondary research pieces. North America as a whole contributed 3.5% (n=4) of the total papers, with a mix of primary and secondary research. Africa and Australia made smaller contributions to the research field, each contributing 1.8% (n=2) of the total papers. In these regions, primary research was minimal, with 1.5% (n=1) each for Africa and Australia, and a small percentage allocated to secondary studies 2.6% (~n=1). This distribution, therefore, highlights a strong research presence in Asia and Europe, where substantial efforts are made in both primary and secondary research components. However, the comparatively lower research output from North America, Africa, and Australia suggests potential areas for increased research focus in future studies, thus increasing the diversity of datasets as shown in Table 4.

**Table 4:** Distribution of papers published in various regions.

Region	Primary, %	Primary and secondary, %	Secondary, %	Total papers published, %
Africa	1.5	0	2.6	1.8
Asia	47.0	66.7	57.9	54.0
Australia	1.5	0	2.6	1.8
Canada	3.0	0	0	1.8
Europe	30.3	16.7	26.3	27.4
North America	3.0	16.7	2.6	3.5
United States	13.6	0	5.3	9.7

### Distribution of Study Designs and Deployment Strategies

A significant proportion of the studies did not implement or reach up to the deployment stage, accounting for 86.4% (n=109) of the total. This high percentage highlights a predominant focus on theoretical or experimental research without necessarily extending to real-world deployment and applications. This consequently portrays a gap in the practical application of the models and methods in infant cry classification systems. Experimental study designs were prominent, comprising 81.7% (n=103) of the reviewed studies, indicating a strong inclination toward hypothesis-driven, controlled investigations. This is consistent with the field’s reliance on experimental setups to validate novel approaches in audio signal processing, particularly for infant cry analysis. The use of primary datasets was reported in 66.7% (n=84) of the studies, depicting the importance of curated, original data in this domain. In comparison, the secondary dataset was used in 36.5% (n=46) of the studies reviewed, demonstrating the occasional reliance on publicly available or previously collected data for further analysis or validation purposes. In terms of specific methods, denoising techniques were performed in 24.6% (n=31) of the studies. This correlates with a lower proportion of studies reaching the production level and reflects the significance of noise reduction in improving the quality and reliability of infant cry signals, which are often affected by environmental noise while in production. Other methodologies included descriptive study designs 15.1% (n=19) and correlational study designs 8.7% (n=11), emphasizing the exploratory nature of some research within the field. These designs aimed to map out underlying relationships and trends within the data but did not necessarily involve the manipulation of variables. However, confidentiality compliance was reported in 7.9% (n=10) of the studies [22-31], pointing to a growing but limited awareness of the ethical considerations in handling sensitive audio data, particularly when it involves infants who are deemed minors. Additionally, quasi-experimental or causal-comparative study designs were used in 7.1% (n=9) of the studies, suggesting that a subset of research explored the causal relationships between variables without random assignment, though this approach was notably less common. Deployment of models in specific platforms was minimal, with software deployment at 4% (n=5) [20,32-35], hardware-based at 4% (n=5) [36-40], Android-based deployment at 2.4% (n=3) [24,40,41], Internet of Things (IoT) integration at 1.6% (n=2) [29,36], and Web deployment at 0.8% (n=1) [25] being among the least used strategies. These findings indicate limited implementation of the developed models in real-world systems, highlighting a critical area for future research in translating experimental findings into practical, scalable solutions and ultimately expanding scientific discoveries at a practical view.

The distribution of application of the deployed models in infant cry classification studies shows clinical diagnosis as being the most prominent at 30.6% (n=39), highlighting a focus on using cry analysis for diagnostic purposes in health care. Hospital-based applications follow at 20.8% (n=26), indicating a strong interest in deploying cry classification in clinical settings to aid in patient monitoring. Home-based care represents 16.7% (n=21), suggesting a practical interest in enabling caregivers to interpret infant cries at home. Infant monitoring systems account for 13.9% (n=17), reflecting use cases in continuous monitoring setups. Research-focused applications make up 11.1% (n=14), emphasizing ongoing exploration and development in the field, while other applications comprise the remaining 6.9% (n=9). The other 6.9% (n=9) category included applications such as wearable technology integration and mobile applications for caregivers. This distribution underscores the versatility of cry classification technology across various settings, from health care facilities to home environments.

### Model Performance and Evaluation

Supervised learning methods achieved the highest recorded accuracy, with a value of 0.9989, confirming the strong performance of supervised models when sufficient labeled data was available for training. Deep learning methods exhibited impressive performance as well, with the maximum accuracy reaching 0.9743. These methods have proven effective in handling complex, high-dimensional cry signals. Transfer learning models demonstrated robust accuracy, with the highest value at 0.9439. The use of pretrained models facilitated significant improvements in classification performance, particularly in cases where data were limited. Probabilistic learning methods recorded a maximum accuracy of 0.9848, indicating their capability to model uncertainties effectively in the cry signal data. However, the consistency of probabilistic learning across different datasets depends on factors such as dataset size, variability, and feature distribution. Unsupervised learning methods reached a maximum accuracy of 0.752, suggesting moderate effectiveness in infant cry classification without labeled data. Other methods, which may include less conventional or hybrid approaches, recorded the lowest maximum accuracy of 0.6743, indicating potential for further refinement or more specialized application in certain contexts.

### Challenges in Infant Cry Modeling

**Figure 5:**
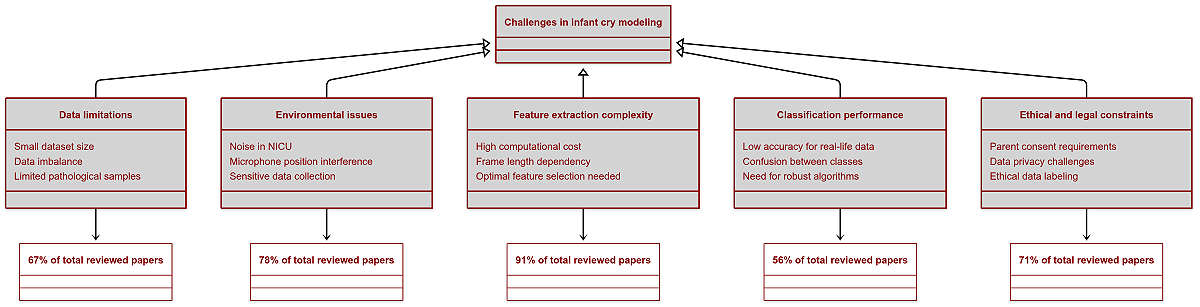
Limitations in infant cry modelling. NICU: neonatal intensive care unit.

The reviewed studies highlighted some of the challenges that researchers experienced in modeling the infant audio cry as depicted in Figure 5. The predominant challenges from the reviewed studies are comprised of data limitations, environmental issues, feature extraction complexity, classification performance, and ethical and legal constraints. The challenges culminated in the adverse impact on development, accuracy, and deployment of infant cry classification models, thus highlighting areas to be explored by the researchers to enhance methodologies and deployment schemes. Data limitations constituted emerged to be one of the challenges from 67% (n=84) of the reviewed studies, pinpointing issues with small dataset sizes, data imbalance, and limited availability of pathological samples. The scarcity of the labeled data, more so from the rare pathological conditions of interest, was realized to be negatively affecting the generalizability of the model and thus necessitates advanced data augmentation methodologies. However, the environmental challenges affected nearly 78% (n=98) of the reviewed studies, mainly due to background noise or within the neonatal intensive care units, which tends to interfere with the microphone placement and sensitivity during data collection processes. The studies showed that ambient noise and microphone variability distorted audio recordings, pausing challenges for models to accurately classify infants’ cry signals. The complexity of the feature extraction of infant audio cries was identified from the 91% (n=115) of the reviewed papers as a technical challenge in modeling of infant needs through audio cries. The process of extraction was associated mainly with the high computational costs and frame length dependency, which in most instances necessitates careful parameter tuning to ensure optimal performance.

Classification performance constraints arose from 56% (n=71) of the reviewed studies with common challenges in attaining high accuracies from models that were deployed into production. From the studies reviewed, it was realized that high performance in controlled environments does not translate to real-world settings, thus presenting a gap in model robustness and the necessity for improved algorithmic approaches with a relatively dynamic generalizability power.

**Ethical and legal constraints** were realized from 71% (n=89) of the studies as major challenges during data collection. These challenges were as a result of parental consent, data privacy, and ethical considerations in data labeling.

## Discussion

### Summary of Findings

The aim of this systematic review piece was to comprehensively analyze the current state of infant cry classification research work, focusing on the methods, trends, and deployment strategies used across different studies. The results indicate a clear preference for machine learning methods, which were applied in over 91% (n=115) of all the studies that were under consideration. This trend, therefore, reflects the continuous adoption of data-driven techniques in infant cry analysis, as machine learning models offer more significant advantages in handling complex and high-dimensional data, such as audio signals in space and time domains. The temporal assessment review pointed to the realization of the substantial application of machine learning gaining upward steady adoptions from the early 2000s to the present day as an audio modeling technique. This evolution highlights a shift from traditional statistical methods, which have seen a notable decline in usage and applications, toward more advanced approaches such as deep learning, transfer learning, and probabilistic learning methods. These trends suggest that the field has embraced modern methodologies, but there remains a gap in the deployment of these models in real-world applications, as indicated by the limited use of platforms such as IoT and Web deployment on the same. Moreover, the geographical exploratory analysis showed that most studies were conducted in Asia and Europe, with Asia accounting for about over 50% (n=63) of the total research output from all studies that were under consideration. This distribution pinpoints the region’s strong research engagement in both foundational and applied studies. However, regions such as North America, Africa, and Australia contributed fewer papers, suggesting potential areas for increased research focus in the future. The performance of the models was largely consistent with expectations. Supervised learning methods were realized to be achieving the highest accuracy, followed closely by deep learning methods, probabilistic learning, and transfer learning models. In contrast, unsupervised learning and other methods showed a notably lower performance, emphasizing the need for further exploration of these approaches in the context of infant cry classification.

### Feature Extraction Methods

Feature extraction plays a pivotal role in signal processing and the infant cry classification process by transforming raw audio signals into relevant attributes and features that can be used to train the model. From the reviewed studies, it was evident that MFCCs were the most used feature across most studies that were under consideration (n=73, 57.9%). MFCCs’ robust ability to capture the perceptual aspects of sound signal, particularly through the representation of the spectral envelope, qualifies them to be highly effective for infant cry discrimination. In extension, this is consistent with the broader field of speech processing, where MFCCs are widely accepted for their potential effectiveness as the foundational representation of both speech and audio signals, thus reflecting inherent ways of human hearing.

Spectrograms were used in 15.9% (n=20) of the studies and emerged as an alternative prominent audio signal feature extraction method. Spectrograms potentially provide a time-frequency representation of the cry audio signal, which enables models, particularly those using convolutional neural networks, to robustly identify patterns and variations in cry characteristics over time. This comprehensively covers both time and space, components forming a strong method for the spatial-temporal domain feature extraction method. The patterns of the infant’s cry signal can be spatially and temporarily represented as either 2 dimensions or 3 dimensions. The notable rise in the use of spectrograms in conjunction with deep learning models highlights a shift toward even more sophisticated, data-driven approaches for infant cry classification. Apart from MFCCs and spectrograms, other commonly used features include pitch, duration, intensity, and 0-crossing rate, all of which tend to provide insights into the acoustic properties of infant cries. These time-domain and frequency-domain features, though less complex than MFCCs or spectrograms, still contribute valuable information to classification tasks, especially in traditional machine learning methods where simpler feature sets are often favored for their interpretability. One notable observation from the studies is the minimal use of wavelet domain features (n=11, 8.7%) [28,42-51]. Wavelet transforms offer the potential to capture both time and frequency information across multiple resolutions, which could enhance the detection of transient events in cry signals, such as sudden changes in pitch or loudness. Despite their theoretical advantages, wavelet-based features have not been as extensively explored, indicating a potential area for future research.

Additionally, the results indicate that prosodic features—such as rhythm, stress, and intonation—were not applied in any of the reviewed studies. Given their importance in speech analysis, prosodic features could be beneficial in understanding the emotional and physiological states conveyed through infant cries [52]. Incorporating prosodic features into future studies may offer deeper insights into the cry patterns associated with different needs or health conditions.

### Implications

The findings of this review offer several important implications for the future of infant cry classification research and shift. The dominance of machine learning techniques indicates that data-driven methods will likely continue to play a pivotal role in advancing the field of applied sciences. However, the moderate performance of unsupervised approaches highlights the need for more research into models that can work with unlabeled data. This is particularly relevant as unsupervised learning methods could prove useful in cases where, for example, large volumes of labeled infant cry data are unavailable or difficult to obtain.

Notably, almost all the proposed models for audio classification are expected to be applied and implemented in a real-world environment where there could be potential background noise. Therefore, the growing use of denoising techniques in 24.6% (n=31) of the studies reflects the significant challenges posed by real-world noise in infant cry audio recordings. As most infant cry recordings are potentially prone to environmental disturbances, future research should focus on developing noise-robust models capable of maintaining high performance without extensive preprocessing. This has been realized in most of the studies showing how small perturbations could affect the precision of the audio signal classification, thus relaxing some of the model parameters.

However, another notable key finding was the limited deployment of these audio classifier models in real-world applications. Despite the advances in model accuracy and precision, very few studies reported actual deployment in mobile or Ios systems, which could bring these novel methodologies closer to practical use in health care or parenting contexts. Researchers and scientists should, therefore, prioritize translating these experimental models into deployable systems that can be tested and validated in real-world environments present with noise sound disturbances.

### Limitations and Challenges Arising From the Reviewed Studies

Challenges arising from classification accuracy under real-life applications show that current algorithms may require enhancements to achieve generalizability from unseen input datasets. Models are preferred to accurately discriminate subtle variations in the infant cries, which potentially reflect different needs or conditions that necessitate more advanced architectures, such as ensemble or multimodal learning frameworks for signal processing and discriminations [53]. The challenges that were encountered in infant cry modeling from the studies reviewed potentially unveil important areas for future innovation and research gaps.

Feature extraction emerged as a primary technical barrier, with high computational requirements and dependencies that may constrain near real-time application solutions [54]. To that extent, optimizing the feature extraction process for efficiency purposes without sacrificing accuracy could be essential for the ultimate deployment of the models in both clinical and parental support applications. For example, empirical approaches such as automated feature learning coupled with adaptive extraction methods could streamline and minimize this challenge [55]. Furthermore, minimizing computational overhead could also smoothen the feature extraction process while enhancing classification precision as the ultimate reward. Background acoustics factors, such as ambient noise and variability in data acquisition, require robust noise mitigation and preprocessing audio methodologies. The infant cries recordings captured in real-world setups, especially in neonatal intensive care units and home environments, are highly susceptible to significant interference where even minor disturbances can significantly impact the audio quality [56]. One way to potentially address this challenge would be to integrate adaptive denoising techniques that can dynamically adjust to various noise conditions as the intermediate layer before the classification stage, which could enhance model reliability and applicability in diverse environments. However, the data constraints observed from the reviewed studies pin the importance of larger, more varied datasets to improve model generalization and reduce overfitting. Collaborative efforts for data-sharing schemes, synthetic data generation, and augmentation could be some of the ways to address these limitations in both quantity and diversity. Ethical and legal considerations, particularly around data privacy and parental consent, are also paramount due to the sensitive nature of infant cry data. Privacy-preserving methods, such as federated learning and differential privacy, could facilitate model development without compromising data security [57]. Standardizing ethical guidelines and consent procedures will not only protect participants but also reinforce public trust in these technologies, essential for broader adoption in health care contexts [58].

### Conclusion

This systematic review has indeed provided a detailed examination of different methods, trends, and strategies used in infant cry classification across different regions in the world. The findings suggest that while supervised learning and deep learning methods currently lead the field in terms of accuracy and performance of audio classification, there is a need for further exploration of other techniques such as unsupervised learning and the ultimate deployment of these models in practical, real-world settings. Moreover, the growing adoption of advanced feature extraction techniques such as MFCCs and spectrograms reflects the increasing sophistication of infant cry analysis models. However, real-world deployment and validation remain limited, pointing to a critical area for future research. By focusing on noise-robust models, unsupervised learning, and practical deployment in health care settings, the field of infant cry classification can continue to evolve and contribute to improved health outcomes for infants. Additionally, there remains an unequal distribution of infant cry classification studies across continents, thus pointing to opportunities for more geographically diverse research. Focusing on these areas will enable continued advancements in infant cry classification, ultimately supporting improved health outcomes for infants and availing audio data for further experimental research.

## Appendix

Multimedia Appendix 1 PRISMA checklist.

Multimedia Appendix 2 PICO framework of the research questions.

Multimedia Appendix 3 Databases and search terms used in the literature search.

Multimedia Appendix 4 TRIPOD checklist for quality assessment of the prediction models.

Multimedia Appendix 5 Cochrane risk of bias. Assessment of the risk of bias for each study.

## Abbreviations

FLACC: Face, Legs, Activity, Cry, Consolability
IoT: Internet of Things
MFCC: Mel-frequency cepstral coefficient
PICO: Problem, Intervention, Comparison, and Outcome
PRISMA: Preferred Reporting Items for Systematic Reviews and Meta-Analyses
RoB: risk of bias
RoB 2: Risk of Bias 2
TRIPOD: Transparent Reporting of a Multivariable Prediction Model for Individual Prognosis or Diagnosis
