# Supplementary material for: Advances in Infant Cry Paralinguistic Classification—Methods, Implementation, and Applications: Systematic Review
Source: JMIR Rehabil Assist Technol. 2025 Apr 29;12:e69457. doi: 10.2196/69457 (PMC12076029; doi:10.2196/69457)
Supplement: Multimedia Appendix 2 [file rehab_v12i1e69457_app2.docx]

| PICO | Concepts | Related Terms for Search |
| --- | --- | --- |
| Problem | Infant Cry Classification and Analysis | "Infant Cry," "Infant Crying," "Baby Cry," "Infant Vocalizations," "Cry Sounds," "Cry Interpretation," "Infant Cry Analysis," "Infant Communication," "Paralinguistic Cues," "Cry Signal Analysis," "Cry Patterns," "Neonatal Vocal Expressions," "Infant State" |
| Medical and Emotional Context |  | "Neonatal Pain," "Hunger Cry," "Distress Vocalization," "Illness Indicator," "Developmental Assessment," "Behavioral Cues," "Psychological Interpretation," "Non-verbal Communication," "Emotional State Detection," "Health Monitoring" |
| Intervention | Advanced Machine Learning and Audio Processing Techniques | "Machine Learning," "Deep Learning," "Neural Networks," "Convolutional Neural Networks (CNN)," "Recurrent Neural Networks (RNN)," "Temporal Convolutional Networks (TCN)," "Support Vector Machines (SVM)," "Random Forests," "Signal Processing," "Acoustic Analysis" |
| Feature Extraction and Audio Representation |  | "Feature Extraction," "Mel-Frequency Cepstral Coefficients (MFCC)," "Spectrogram," "Auditory Features," "Signal Processing Techniques," "Temporal Features," "Frequency Features," "Waveform Analysis," "Audio Classification Techniques," "Speech Signal Analysis" |
