# Supplementary material for: Advances in Infant Cry Paralinguistic Classification—Methods, Implementation, and Applications: Systematic Review
Source: JMIR Rehabil Assist Technol. 2025 Apr 29;12:e69457. doi: 10.2196/69457 (PMC12076029; doi:10.2196/69457)
Supplement: Multimedia Appendix 3 [file rehab_v12i1e69457_app3.docx]

| **Database** | **Search Terms** | **Publications Retrieved** |
| --- | --- | --- |
| **Google Scholar** | (“Infant Cry” OR “Infant Crying” OR “Baby Cry” OR “Infant Vocalizations”) AND (“Machine Learning” OR “Neural Networks” OR “Signal Processing” OR “Auditory Analysis” OR “Spectrogram”) AND (“Accuracy” OR “Deployment” OR “Application” OR “Real-Time Analysis”) | 1,484 |
| **IEEE Xplore** | (“Infant Cry” OR “Infant Crying” OR “Baby Cry” OR “Infant Vocalizations”) AND (“Machine Learning” OR “Deep Learning” OR “Signal Processing” OR “Neural Networks” OR “Spectrogram”) AND (“Accuracy” OR “Deployment” OR “Application” OR “Real-Time Analysis”) | 325 |
| **PubMed** | (“Infant Cry” [MESH] OR “Infant Crying” OR “Baby Cry” OR “Infant Vocalizations”) AND (“Machine Learning” OR “Neural Networks” OR “Signal Processing” OR “Auditory Analysis” OR “Spectrogram”) AND (“Accuracy” OR “Deployment” OR “Application” OR “Real-Time Analysis”) | 550 |
| **Scopus** | (“Infant Cry” OR “Infant Crying” OR “Baby Cry” OR “Infant Vocalizations”) AND (“Machine Learning” OR “Neural Networks” OR “Signal Processing” OR “Auditory Analysis” OR “Spectrogram”) AND (“Accuracy” OR “Deployment” OR “Application” OR “Real-Time Analysis”) | 800 |
| **Web of Science Core Collection** | (“Infant Cry” OR “Infant Crying” OR “Baby Cry” OR “Infant Vocalizations”) AND (“Machine Learning” OR “Neural Networks” OR “Signal Processing” OR “Auditory Analysis” OR “Spectrogram”) AND (“Accuracy” OR “Deployment” OR “Application” OR “Real-Time Analysis”) | 625 |
| **MEDLINE** | (“Infant Cry” OR “Infant Crying” OR “Baby Cry” OR “Infant Vocalizations”) AND (“Machine Learning” OR “Neural Networks” OR “Signal Processing” OR “Auditory Analysis” OR “Spectrogram”) AND (“Accuracy” OR “Deployment” OR “Application” OR “Real-Time Analysis”) | 400 |
| **Cochrane Database of Systematic Reviews (CDSR)** | (“Infant Cry” OR “Infant Crying” OR “Baby Cry” OR “Infant Vocalizations”) AND (“Machine Learning” OR “Neural Networks” OR “Signal Processing” OR “Auditory Analysis” OR “Spectrogram”) AND (“Accuracy” OR “Deployment” OR “Application” OR “Real-Time Analysis”) | 550 |
| **JSTOR** | (“Infant Cry” OR “Infant Crying” OR “Baby Cry” OR “Infant Vocalizations”) AND (“Machine Learning” OR “Deep Learning” OR “Neural Networks” OR “Signal Processing” OR “Auditory Analysis”) AND (“Classification Accuracy” OR “Practical Applications” OR “Deployment”) | 670 |
| **ACM Digital Library** | (“Infant Cry” OR “Infant Crying” OR “Baby Cry” OR “Infant Vocalizations”) AND (“Machine Learning” OR “Neural Networks” OR “Signal Processing” OR “Auditory Analysis” OR “Spectrogram”) AND (“Accuracy” OR “Deployment” OR “Application” OR “Real-Time Analysis”) | 500 |
| **TOTAL** |  | **5,904** |
