# Supplementary material for: Advances in Infant Cry Paralinguistic Classification—Methods, Implementation, and Applications: Systematic Review
Source: JMIR Rehabil Assist Technol. 2025 Apr 29;12:e69457. doi: 10.2196/69457 (PMC12076029; doi:10.2196/69457)
Supplement: Multimedia Appendix 5 [file rehab_v12i1e69457_app5.docx]

| Author | Title | Randomization Process | Deviation from Intended Outcome | Missing Outcome Data | Selection of Reported Results | Measurement of Outcome | RoB Overall |
| --- | --- | --- | --- | --- | --- | --- | --- |
| Krittakom Srijiranon, Narissara Eiamkanitchat | Application of Neuro-fuzzy approaches to recognition and classification of infant cry | Low risk | Low risk | Low risk | Low risk | Low risk | Low risk |
| Seyyedeh Fatemeh Molaeezadeh, Mehrnoosh Salarian, Mohammad Hassan Moradi | Type-2 Fuzzy Pattern Matching for Classifying Hunger and Pain Cries of Healthy Full-term Infants | Low risk | Low risk | Low risk | Low risk | Low risk | Low risk |
| Lillian Le, Abu Nadim M.H. Kabir, Chunyan Ji, Sunitha Basodi, Yi Pan | Using Transfer Learning, SVM, and Ensemble Classification to classify Baby Cries based on their Spectrogram Images | Low risk | Low risk | Low risk | Low risk | Low risk | Low risk |
| MARCO SEVERINI, DANIELE FERRETTI, EMANUELE PRINCIPI , AND STEFANO SQUARTIN | Automatic Detection of Cry Sounds in NICUs by Using Deep Learning and Acoustic Scene Simulation | Low risk | Low risk | Some concerns | Low risk | Low risk | Low risk |
| M. Z. Mohd Ali, W. Mansor, Y. K. Lee, A. Zabidi | Asphyxiated Infant Cry Classification Using Simulink Model | Low risk | Low risk | Low risk | Low risk | Low risk | Low risk |
| Azlee Zabidi, Wahidah Mansor, Lee Yoot Khuan, Ihsan Mohd Yassin, Rohilah Sahak | Binary Particle Swarm Optimization for Selection of Features in the Recognition of Infants Cries with Asphyxia | Low risk | Low risk | Low risk | Low risk | Low risk | Low risk |
| Azlee Zabidi, Lee Yoot Khuan, Wahidah Mansor, Ihsan Mohd Yassin, Rohilah Sahak | Detection of Infant Hypothyroidism with Mel Frequency Cepstrum Analysis and Multi-Layer Perceptron Classification | Low risk | Low risk | Low risk | Low risk | Low risk | Low risk |
| R. ROBU, F. FEIER, V. STOICU-TIVADAR, C. ILIE and I. EN | The analysis of the new-borns’ cry using NEONAT and data mining techniques | Low risk | Low risk | Some concerns | Low risk | Some concerns | Some concerns |
| Rodica Ileana TUDUCE*, Mircea Sorin RUSU†, Horia CUCU*, and Corneliu BURILEANU | Automated Baby Cry Classification on a Hospital-acquired Baby Cry Database | Low risk | Low risk | Low risk | Low risk | Low risk | Low risk |
| Aomar Osmani,Massinissa Hamidi and Abdelghani Chibani | Machine Learning Approach for Infant Cry Interpretation | Low risk | Low risk | Low risk | Low risk | Low risk | Low risk |
| Mohammad Kia, Shabnam Kia, Neda Davoudi, Ramyar Biniazan | A Detection System of Infant Cry Using Fuzzy Classification Including Dialing Alarm Calls Function | Low risk | Low risk | Low risk | Low risk | Low risk | Low risk |
| HesamFarsaieAlaie,LinaAbou-Abbas,ChakibTadj | Cry-based infant pathology classification using GMMs | Low risk | Low risk | Low risk | Low risk | Low risk | Low risk |
| Ioana-Alina B nic , Horia Cucu*, Andi Buzo, Drago Burileanu and Corneliu Burileanu | Automatic Methods for Infant Cry Classification | Low risk | Low risk | Low risk | Low risk | Low risk | Low risk |
| Avinash Kumar Singh, Jayanta Mukhopadhyay and K. Sreenivasa Rao | CLASSIFICATION OF INFANT CRIES USING SOURCE, SYSTEM AND SUPRA-SEGMENTAL FEATURES | Low risk | Some concerns | Low risk | Low risk | Low risk | Some concerns |
| Rami Cohen, YizharLavner | Infant Cry Analysi and Detection | Low risk | Some concerns | Low risk | Low risk | Low risk | Low risk |
| Chuan-Yu Chang, Yu-Chi Hsiao,Szu-Ta Chen | APPLICATION OF INCREMENTAL SVM LEARNING FOR INFANT CRIES RECOGNITION | Low risk | Low risk | Low risk | Low risk | Low risk | Low risk |
| Omnia Magdy Badreldine, Nourhan A. Elbeheiry, Ahmad Nashaat M. Haroon, Saleh ElShehaby | Automatic Diagnosis of Asphyxia Infant Cry Signals Using Wavelet Based Mel Frequency Cepstrum Features | Low risk | Low risk | Low risk | Some concerns | Low risk | Low risk |
| Karinki Manikanta,K.P. Soman,M. Sabarimalai Manikandan | Deep Learning Based Effective Baby Crying Recognition Method under Indoor Background Sound Environments | Low risk | Low risk | Low risk | Low risk | Low risk | Low risk |
| GustavoZ. Felipe, Rafael L. Aguiar, Yandre M. G. Costa, Carlos N. Siila Jr., Sheryl Brahnam3 Loris Nanni, Shannon McMurtrey5 | Identification of Infants’ Cry Motivation Using Spectrograms | Low risk | Low risk | Low risk | Low risk | Low risk | Low risk |
| Garvit Joshi, Hardik Tiwari,Chaitanya Dandvate, Aakash Mundhare | Prediction of Probability of Crying of A Child and System Formation for Cry Detection and Financial Viability of the System | Low risk | Low risk | Low risk | Low risk | Low risk | Low risk |
| Yara Zayed,AhmadHasasneh1,and Chakib Tadj | Infant Cry Signal Diagnostic System Using Deep Learning and Fused Features | Low risk | Low risk | Low risk | Low risk | Low risk | Low risk |
| STAVROS NTALAMPIRAS | Audio Pattern Recognition of Baby Crying Sound Events | Low risk | Low risk | Low risk | Low risk | Low risk | Low risk |
| Mark Huckvale | Neural network architecture that combines temporal and summative features for infant cry classification in the Interspeech 2018 Computational Paralinguistics Challenge | Low risk | Low risk | Low risk | Low risk | Low risk | Low risk |
| Karen Santiago-Sánchez, Carlos A. Reyes-García, and Pilar Gómez-Gil | Type-2 Fuzzy Sets Applied to Pattern Matching for the Classification of Cries of Infants under Neurological Risk | Low risk | Low risk | Low risk | Low risk | Low risk | Low risk |
| Tusty Nadia Maghfira et al | Infant cry classification using CNN – RNN | Low risk | Low risk | Low risk | Low risk | Low risk | Low risk |
| Lichuan Liu, Yang Li, Kevin Kuo | Infant Cry Signal Detection, Pattern Extraction and Recognition | Low risk | Low risk | Low risk | Low risk | Low risk | Low risk |
| Sita Purnama Dewi,Anggunmeka Luhur Prasasti ,Budhi Irawan | The Study of Baby Crying Analysis Using MFCC and LFCC in Different Classification Methods | Low risk | Low risk | Low risk | Low risk | Low risk | Low risk |
| Alejandro Rosales-Péreza, Carlos A. Reyes-Garcíaa, Jesus A. Gonzaleza, Orion F. Reyes-Galavizb, Hugo Jair Escalantea, Silvia Orlandic | Classifying infant cry patterns by the Genetic Selection of a Fuzzy Model | Low risk | Low risk | Low risk | Low risk | Low risk | Low risk |
| Kushal Sharma,Chirag Gupta,Dr. Sandeep Gupta | Infant Weeping Calls Decoder using Statistical Feature Extraction and Gaussian Mixture Models | Low risk | Low risk | Low risk | Low risk | Low risk | Low risk |
| Mrs. Sameena Bano,Dr. K.M. RaviKumar, | Decoding Baby Talk: A Novel Approach for Normal Infant Cry Signal Classification | Low risk | Low risk | Low risk | Low risk | Low risk | Low risk |
| Yizhar Lavner, Rami Cohen, Dima Ruinskiy∗‡ and Hans IJzerman§ | Baby Cry Detection in Domestic Environment using Deep Learning | Low risk | Low risk | Low risk | Low risk | Low risk | Low risk |
| Silvia Orlandi, †Carlos Alberto Reyes Garcia,‡Andrea Bandini, §Gianpaolo Donzelli, and Claudia Manfredi, | Application of Pattern Recognition Techniques to the Classification of Full-Term and Preterm Infant Cry | Low risk | Low risk | Low risk | Low risk | Low risk | Low risk |
| D Widhyanti and DJuniati | Classification of Baby Cry Sound Using Higuchi’s Fractal Dimension with K-Nearest Neighbor and Support Vector Machine | Low risk | Low risk | Low risk | Low risk | Some concerns | Some concerns |
| Anyawee Chaiwachiragompol, Nattawoot Suwannata | The Study of Learning System for Infant Cry Classification Using Discrete Wavelet Transform and Extreme Machine Learning | Low risk | Low risk | Low risk | Low risk | Low risk | Low risk |
| Vinayak Ravi Joshi, Kathiravan Srinivasan, P. M. Durai Raj Vincent, Venkatesan Rajinikanth and Chuan-Yu Chang | A Multistage Heterogeneous Stacking Ensemble Model for Augmented Infant Cry Classification | Low risk | Low risk | Low risk | Low risk | Low risk | Low risk |
| Yizhar Lavner , Rami Cohen , Dima Ruinskiy and Hans IJzerman | Baby Cry Detection in Domestic Environment using Deep Learning | Low risk | Low risk | Low risk | Low risk | Low risk | Low risk |
| Chunyan Ji, Ming Chen, Bin Li, Yi Pan | Infant Cry Classification with Graph Convolutional Networks | Low risk | Low risk | Low risk | Low risk | Low risk | Low risk |
| Ashwini K, P. M. Durai Raj Vincent, Kathiravan Srinivasan and Chuan-Yu Chang | Deep Learning Assisted Neonatal Cry Classification via Support Vector Machine Models | Low risk | Low risk | Low risk | Low risk | Low risk | Low risk |
| Azadeh Bashiri, Roghaye Hosseinkhani | Infant Crying Classification by Using Genetic Algorithm and Artificial Neural Network | Low risk | Low risk | Low risk | Low risk | Some concerns | Low risk |
| Anamaria Radoi and Corneliu Burileanu | Infant Cry Classification Using Compression-Based Similarity Metric | Low risk | Low risk | Low risk | Low risk | Some concerns | Low risk |
| Takayuki Kurokawa, Tasuku Miura, Masaru Yamashita, Tomoya Sakai and Shoichi Matsunaga | Emotion-Cluster Classification of Infant Cries Using Sparse Representation | Low risk | Low risk | Low risk | Low risk | Low risk | Low risk |
| Leandro D. Vignolo(B), Enrique Marcelo Albornoz ,and C´ esar Ernesto Mart´ ınez | Feature Set Optimisation for Infant Cry Classification | Low risk | Low risk | Some concerns | Low risk | Low risk | Some concerns |
| Yosra Abdulaziz Mohammed, | Infant Cry Recognition System: A Comparison of System Performance based on CDHMM and ANN | Low risk | Low risk | Low risk | Low risk | Low risk | Low risk |
| Alishamol K. S., Fousiya T. T., Jasmin Babu K., Sooryadas M., and Leena Mary | System for Infant Cry Emotion Recognition using DNN | Low risk | Low risk | Low risk | Low risk | Low risk | Low risk |
| Chunyan Ji,Xueli Xiao ,Sunitha Basodi and Yi Pan | Deep Learning for Asphyxiated Infant Cry Classification Based on Acoustic Features and Weighted Prosodic Features | Low risk | Low risk | Low risk | Low risk | Low risk | Low risk |
| K. Teeravajanadet1, N. Siwilai2, K. Thanaselanggul3, N. Ponsiricharoenphan4, S. Tungjitkusolmun5, P. Phasukkit6 | An Infant Cry Recognition based on Convolutional Neural Network Method | Low risk | Low risk | Low risk | Low risk | Low risk | Low risk |
| Xilin Yu et al. | An Investigation into Audio Features and DTW Algorithms for Infant Cry Classification | Low risk | Low risk | Low risk | Low risk | Low risk | Low risk |
| Atal Sharma, Dr. Deepti Malhotra | Speech recognition based IICC - Intelligent Infant Cry Classifier | Low risk | Low risk | Low risk | Some concerns | Low risk | Low risk |
| Prathamesh Kulkarniet al. | Child Cry Classification - An Analysis of Features and Models | Low risk | Low risk | Low risk | Low risk | Low risk | Low risk |
| Ashwini K, Durai Raj Vincent P M, Kathiravan Srinivasan and Chuan-Yu Chang | Deep Convolutional Neural Network based Feature Extraction with optimized Machine Learning Classifier in Infant Cry Classification | Low risk | Low risk | Low risk | Low risk | Low risk | Low risk |
| Amany Mounes Mahmoud, Sarah Mohamed Swilem, Abrar Saeed Alqarni, Fazilah Haron | Infant Cry Classification Using Semi-supervised K-Nearest Neighbor Approach | Low risk | Low risk | Low risk | Low risk | Low risk | Low risk |
| Matheus H. C. Sudul1, Rafael L. Aguiar1, Yandre M. G. Costa2, Sheryl Brahnam3, Shannon McMurtrey4, Loris Nanni5 and Carlos N. Silla Jr.1 | Automatic Classification of Infant’s Cry Using Data Balancing and Hierarchical Classification Techniques | Low risk | Some concerns | Low risk | Low risk | Low risk | Some concerns |
| Chuan-Yu Chang,Sweta Bhattacharya  Kuruva Lakshmanna, 3P.M.Durai Raj Vincent, and Kathiravan Srinivasan | An Efficient Classification of Neonates Cry Using Extreme Gradient Boosting-Assisted Grouped-Support-Vector Network. | Low risk | Low risk | Low risk | Low risk | Low risk | Low risk |
| Aastha Kachhi, Shreya Chaturvedi, Hemant A. Patil, Dipesh Kumar Singh | Data Augmentation for Infant Cry Classification | Low risk | Low risk | Low risk | Low risk | Low risk | Low risk |
| KHOSRO REZAEE, HOSSEIN GHAYOUMI ZADEH, LIANYONG QI ,HAMIDREZA RABIEE, MOHAMMAD R. KHOSRAVI | Can You Understand Why I Am Crying? A Decision-making System for Classifying Infants’ Cry Languages Based on DeepSVM Model | Low risk | Low risk | Low risk | Low risk | Low risk | Low risk |
| Ankur T. Patil, Aastha Kachhi, Hemant A. Patil | Subband Teager Energy Representations for Infant Cry Analysis and Classification | Low risk | Low risk | Low risk | Low risk | Low risk | Low risk |
| Aastha Kachhi, Priyanka Gupta, Hemant A. Patil | Features Motivated From Uncertainty Principle for Classification of Normal vs. Pathological Infant Cry | Low risk | Low risk | Low risk | Low risk | Low risk | Low risk |
| Chunyan Ji, Yi Pan | Infant Vocal Tract Development Analysis and Diagnosis by Cry Signals with CNN Age Classification | Some concerns | Low risk | Low risk | Low risk | Low risk | Low risk |
| Hardik B. Sailor, Hemant Patil | Auditory Filterbank Learning Using ConvRBM for Infant Cry Classification. | Low risk | Low risk | Low risk | Low risk | Low risk | Low risk |
| Ramon L. Rodriguez and Susan S. Caluya | Infants Cry Classification of Physiological State Using Cepstral and Prosodic Acoustic Features | Low risk | Low risk | Low risk | Low risk | Low risk | Low risk |
| Wei Jer Lim, Hariharan Muthusamy, Vikneswaran Vijean, Haniza Yazid Thiyagar Nadarajaw3, Sazali Yaacob | Dual-Tree Complex Wavelet Packet Transform and Feature Selection Techniques for Infant Cry Classification | Low risk | Low risk | Low risk | Low risk | Low risk | Low risk |
| Aditya Pusuluri(B), Aastha Kachhi, and Hemant A. Patil | Analysis of Time-Averaged Feature Extraction Techniques on Infant Cry Classification | Low risk | Low risk | Low risk | Low risk | Low risk | Low risk |
| J. Saraswathy Q1 , M. Hariharanb, Wan Khairunizama, J. Sarojinic, N. Thiyagard, Y. Sazalie, Shafriza Nisha | Time–frequency analysis in infant cry classification using quadratic time frequency distributions | Low risk | Low risk | Low risk | Low risk | Low risk | Low risk |
| Avinash Kumar Singh, Jayanta Mukhopadhyay and K. Sreenivasa Rao | Classification of Infant Cries Using Epoch and Spectral Features | Low risk | Low risk | Low risk | Low risk | Low risk | Low risk |
| D. Ricossa; E. Baccaglini2; E. Di Nardo; E. Parodi3; R. Scopigno2 | On the automatic audio analysis and classification of cry for infant pain assessment | Low risk | Low risk | Low risk | Low risk | Low risk | Low risk |
| Gianpaolo Coro1 •;Serena Bardelli2;Armando Cuttano; Rosa T. Scaramuzzo; Massimiliano Ciantelli | A self-training automatic infant-cry detector | Low risk | Low risk | Low risk | Low risk | Low risk | Low risk |
| Yun-Chia Liang , Iven Wijaya , Ming-Tao Yang , Josue Rodolfo Cuevas Juarez 1 and Hou-Tai Chang | Deep Learning for Infant Cry Recognition | Low risk | Low risk | Low risk | Low risk | Low risk | Low risk |
| Salim Lahmiri, Chakib Tadj, and Christian Gargour | Biomedical Diagnosis of Infant Cry Signal Based on Analysis of Cepstrum by Deep Feedforward Artificial Neural Networks | Low risk | Low risk | Low risk | Low risk | Low risk | Low risk |
| Anshu Chittora;· Hemant A. Patil | Significance of Higher-Order Spectral Analysis in Infant Cry Classification | Low risk | Low risk | Low risk | Low risk | Low risk | Low risk |
| Shane Grayson1 and Wilson Zhu2 | BABY CRY CLASSIFICATIONS USING DEEP LEARNING | Low risk | Low risk | Low risk | Low risk | Low risk | Low risk |
| Avinash Kumar Singh, Jayanta Mukhopadhyay, Sunil Kumar S B, and K. Sreenivasa Ra | Infant Cry Recognition using Excitation Source Features | Low risk | Low risk | Low risk | Low risk | Low risk | Low risk |
| Lichuan Liu, Kevin Kuo and Sen M. Kuo | Infant Cry Classification Integrated ANC System for Infant Incubators | Low risk | Low risk | Low risk | Low risk | Low risk | Low risk |
| N.S.A.Wahid, P.Saad, M.Hariharan | Automatic Infant Cry Pattern Classification for a Multiclass Problem | Low risk | Low risk | Low risk | Low risk | Low risk | Low risk |
| Boon Fei Yong, Hua Nong Ting, and Kwan Hoong Ng | Baby Cry Recognition Using Deep Neural Networks | Low risk | Low risk | Low risk | Low risk | Low risk | Low risk |
| Yesy Diah Rosita;Hartarto Junaedi | Infant’s Cry Sound Classification using Mel- Frequency Cepstrum Coefficients Feature Extraction and Backpropagation Neural Network | Low risk | Low risk | Low risk | Low risk | Low risk | Low risk |
| Rodica Ileana TUDUCE, Mircea Sorin RUSU, Horia CUCU, and Corneliu BURILEANU | Automated Baby Cry Classification on a Hospital-acquired Baby Cry Database | Low risk | Low risk | Low risk | Low risk | Low risk | Low risk |
| Daniele Ferretti, Marco Severini, Emanuele Principi, Annalisa Cenci, and Stefano Squartini | Infant Cry Detection in Adverse Acoustic Environments by Using Deep Neural Networks | Low risk | Low risk | Low risk | Low risk | Low risk | Low risk |
| Xuewen Yao, Megan Micheletti, Mckensey Johnson,Kaya de Barbaro | Classification of Infant Crying in Real-World Home Environments Using Deep Learning | Low risk | Some concerns | Low risk | Low risk | Low risk | Some concerns |
| Chuan-Yu Chang, Chuan-Wang Chang, S. Kathiravan, Chen Lin & Szu-Ta Chen | DAG-SVM based infant cry classification system using sequential forward floating feature selection | Low risk | Low risk | Low risk | Low risk | Low risk | Low risk |
| Shubham Asthana,Naman Varma,Vinay Kumar Mittal | An Investigation into Classification of Infant Cries  using Modified Signal Processing Methods | Low risk | Low risk | Low risk | Low risk | Low risk | Low risk |
| Orion Fausto Reyes-Galaviz, Sergio Daniel Cano-Ortiz, Carlos Alberto Reyes-Garc´ıa | Evolutionary-Neural System to Classify Infant Cry Units for Pathologies Identification in Recently Born Babies | Low risk | Low risk | Low risk | Low risk | Low risk | Low risk |
| Dror Lederman', Arnon Cohen, Ehud'Zmora , Kathleen Wermke , Stephanie Ha~schildt-~  and Angelika Stellzig-Eisenhauer | On the Use of Hidden Markov Models in Infants' Cry Classification | Low risk | Low risk | Low risk | Low risk | Low risk | Low risk |
| Orion Fausto Reyes Galaviz and Carlos Alberto Reyes Garcia | Infant Cry Classification to Identify Hypoacoustics and Asphyxia with Neural Networks | Low risk | Low risk | Low risk | Low risk | Low risk | Low risk |
| Pritam Pal, Ananth N. Iyer and Robert E. Yantorno, | EMOTION DETECTION FROM INFANT FACIAL EXPRESSIONS AND CRIES | Low risk | Low risk | Low risk | Low risk | Low risk | Low risk |
| Sergio D. Cano Ortiz, Daniel. Escobedo Beceiro, and Taco Ekkel | A Radial Basis Function Network Oriented  for Infant Cry Classification | Some concerns | Low risk | Low risk | Low risk | Low risk | Some concerns |
| Erika Amaro-Camargo and Carlos A. Reyes-García | Applying Statistical Vectors of Acoustic Characteristics  for the Automatic Classification of Infant Cry | Low risk | Low risk | Low risk | Low risk | Low risk | Low risk |
| Kevin Kuo | Feature Extraction and Recognition of Infant Cries | Low risk | Low risk | Low risk | Low risk | Low risk | Low risk |
| Azlee Zabidi, Lee Yoot Khuan, Wahidah Mansor, Ihsan Mohd Yassin, Rohilah Sahak | Classification of Infant Cries with Asphyxia Using Multilayer Perceptron Neural Network | Low risk | Low risk | Low risk | Low risk | Low risk | Low risk |
| Orion F. Reyes-Galaviz, Antonio Verduzco,  Emilio Arch-Tirado, and Carlos A. Reyes-García3 | Analysis of an Infant Cry Recognizer  for the Early Identification of Pathologies | Low risk | Low risk | Low risk | Low risk | Low risk | Low risk |
| Azlee Zabidi, Wahidah Mansor, Lee Yoot Khuan, Ihsan Mohd Yassin, Rohilah Sahak | Classification of Infant Cries with Hypothyroidism  Using Multilayer Perceptron Neural Network | Low risk | Low risk | Low risk | Low risk | Low risk | Low risk |
| R. Sahak, W. Mansor, Y. K. Lee, A. I Mohd Yassin, A. Zabidi | Orthogonal Least Square Based Support Vector  Machine for the Classification of Infant Cry with  Asphyxia | Low risk | Low risk | Low risk | Low risk | Low risk | Low risk |
| Jose Orozco Garcia, Carlos A. Reyes Garcia | Mo-frequency cepstrum coefficients extraction from infant cry for classification of normal and pathological cry with feed-forward neural networks | Low risk | Some concerns | Low risk | Some concerns | Low risk | Some concerns |
| Dror Lederman Æ Ehud Zmora Æ Stephanie Hauschildt Æ  Angelika Stellzig-Eisenhauer Æ Kathleen Wermk | Classification of cries of infants with cleft-palate using parallel  hidden Markov model | Low risk | Low risk | Low risk | Low risk | Low risk | Low risk |
| Hemant A. Patil | “Cry Baby”: Using Spectrographic Analysis  to Assess Neonatal Health Status  from an Infant’s Cry | Low risk | Low risk | Low risk | Low risk | Low risk | Low risk |
| Orion F. Reyes-Galaviz* & Carlos Alberto Reyes-Garcia** | A System for the Processing of Infant Cry to Recognize Pathologies in Recently  Born Babies with Neural Networks | Low risk | Low risk | Some concerns | Low risk | Low risk | Low risk |
| José Orozco, Carlos A. Reyes García | Detecting Pathologies from Infant Cry Applying Scaled Conjugated Gradient  Neural Networks | Low risk | Low risk | Low risk | Low risk | Low risk | Low risk |
| R. Sahak, W. Mansor, Y. K. Lee, A. I. M. Yassin, A. Zabidi | Performance of Combined Support Vector Machine and Principal  Component Analysis in Recognizing Infant Cry with Asphyxia | Low risk | Low risk | Low risk | Low risk | Low risk | Low risk |
| H.E. Baeck, M. N. Souza | A Bayesian Classifier for Baby’s Cry in Pain and Non-pain Contexts | Low risk | Low risk | Low risk | Low risk | Low risk | Low risk |
| Claudia Manfredi, Member, Valentina Tocchioni, Leonardo Bocchi | A Robust Tool for Newborn Infant Cry Analysis | Low risk | Low risk | Low risk | Low risk | Low risk | Low risk |
| Sandra E. Barajas-Montiel,Carlos A. Reyes-García | Identifying Pain and Hunger in Infant Cry with Classifiers Ensembles | Low risk | Low risk | Low risk | Low risk | Low risk | Low risk |
| José Orozco-García and Carlos A. Reyes-García | A Study on the Recognition of Patterns of Infant Cry for  the Identification of Deafness in Just Born Babies with  Neural Network | Low risk | Some concerns | Low risk | Low risk | Some concerns | Some concerns |
| Israel Suaste-Rivas, Alejandro Díaz-Méndez, Carlos A. Reyes-García, and Orion F. Reyes-Galaviz | Hybrid Neural Network Design and Implementation on FPGAfor Infant Cry Recognition | Low risk | Low risk | Some concerns | Low risk | Low risk | Low risk |
| A. Zabidi, W. Mansor, Y. K. Lee, A. I. Mohd Yassin, R. Sahak | Particle Swarm Optimisation of Mel-frequency  Cepstral Coefficients Computation for the  Classification of Asphyxiated Infant Cry | Low risk | Low risk | Low risk | Low risk | Low risk | Low risk |
| R. Sahak, Y. K. Lee, W. Mansor, A. I. M. Yassin, A. Zabidi | Optimized Support Vector Machine for Classifying  Infant Cries with Asphyxia using Orthogonal Least  Square | Low risk | Low risk | Low risk | Low risk | Low risk | Low risk |
| MANNES POEL, TACO EKKEL | ANALYZING INFANT CRIES USING A COMMITTEE OF NEURAL NETWORKS IN ORDER TO DETECT HYPOXIA RELATED DISORDER∗, | Low risk | Low risk | Low risk | Low risk | Low risk | Low risk |
| C. Manfredia,∗, L. Bocchia, S. Orlandia, L. Spaccaterrab, G.P. Donzelli | High-resolution cry analysis in preterm newborn infants | Low risk | Low risk | Low risk | Low risk | Low risk | Low risk |
| Sheryl Brahnam1, Loris Nanni2, Randall Sexton1 | Introduction to Neonatal Facial Pain Detection Using Common and Advanced Face Classification Techniques | Low risk | Low risk | Low risk | Low risk | Low risk | Low risk |
| S. Matsunaga, S. Sakaguchi1, M. Yamashita, S. Miyahara, S. Nishitani and K. Shinohara | Emotion Detection in Infants’ Cries Based on a Maximum Likelihood Approach | Low risk | Low risk | Low risk | Low risk | Low risk | Low risk |
| J. OROZCO-GARCÍA, CARLOS A. REYES-GARCÍA | Applying Scaled Conjugate Gradient for the Classification of Infant Cry  with Neural Networks | Low risk | Low risk | Low risk | Low risk | Low risk | Low risk |
| J. Saraswathy, M. Hariharan, Vikneswaran Vijean, Sazali Yaacob and Wan Khairunizam | Performance Comparison of Daubechies Wavelet  Family in Infant Cry Classification | Low risk | Low risk | Low risk | Low risk | Low risk | Low risk |
| J. Saraswathy ,M. Hariharan , Thiyagar Nadarajaw, Wan Khairunizam , Sazali Yaacob | Optimal selection of mother wavelet for accurate infant cry classification | Low risk | Low risk | Low risk | Low risk | Low risk | Low risk |
| Anshu Chittora and Hemant A. Patil | Classification of Pathological Infant Cries using Modulation Spectrogram  Features | Low risk | Low risk | Some concerns | Low risk | Some concerns | Some concerns |
| M. Hariharan⇑, Sazali Yaacob, Saidatul Ardeenaawatie Awang | Pathological infant cry analysis using wavelet packet transform  and probabilistic neural network | Low risk | Low risk | Low risk | Low risk | Low risk | Low risk |
| Anshu Chittora and Hemant A. Patil | CLASSIFICATION OF NORMAL AND PATHOLOGICAL INFANT CRIES USING  BISPECTRUM FEATURES | Low risk | Low risk | Low risk | Low risk | Low risk | Low risk |
| HesamFarsaieAlaieandChakibTadj | Cry-Based Classification of Healthy and Sick  Infants Using Adapted Boosting MixtureLearningMethodfor  Gaussian Mixture Models | Some concerns | Low risk | Low risk | Low risk | Low risk | Some concerns |
| M. Hariharan & Lim Sin Chee & Sazali Yaacob | Analysis of Infant Cry Through Weighted Linear Prediction  Cepstral Coefficients and Probabilistic Neural Network | Low risk | Low risk | Low risk | Low risk | Low risk | Low risk |
| Tanja Etz ,Henning Reetz ,Carla Wegener | A Classification Model for Infant Cries  with Hearing Impairment and Unilateral  Cleft Lip and Palate | Low risk | Low risk | Low risk | Low risk | Low risk | Low risk |
| Biswanath Saha , Parimal Kumar Purkait , Jayanta Mukherjee ,Arun Kumar Majumdar , Bandana Majumdar and Arun Kumar Singh | An Embedded System for Automatic Classification of Neonatal Cry | Low risk | Some concerns | Low risk | Low risk | Low risk | Low risk |
| A. Zabidi1, W. Mansor2, L. Y. Khuan, I. M. Yassin, R. Sahak | The effect of F-Ratio in the Classification of  Asphyxiated Infant Cries Using Multilayer Perceptron  Neural Network | Low risk | Low risk | Low risk | Low risk | Low risk | Low risk |
| K. Kitahara1, S. Michiwiki1, M. Sato1, S. Matsunaga1, M. Yamashita1 and K. Shinohara2 | Emotion Classification of Infants’ Cries using Duration Ratios of Acoustic  Segments | Low risk | Low risk | Low risk | Low risk | Low risk | Low risk |
| Lina Abou-Abbas, Chakib Tadj, and Hesam Alaie Fersaie | A fully automated approach for baby cry signal segmentation and boundary detection of expiratory and inspiratory episodes | Low risk | Low risk | Low risk | Low risk | Low risk | Low risk |
| Natlada Meephiw; Pakorn Leesutthipornchai | MFCC Feature Selection for Infant Cry Classification | Low risk | Low risk | Low risk | Low risk | Low risk | Low risk |
| Golla Anjali; Santosh Sanjeev; Akuraju Mounika; Gangireddy Suhas; G. Pradeep Reddy | Infant Cry Classification using Transfer Learning | Low risk | Low risk | Low risk | Low risk | Low risk | Low risk |
| Hemant A. Patil; Ankur T. Patil; Aastha Kachhi | Constant Q Cepstral coefficients for classification of normal vs. Pathological infant cry | Low risk | Low risk | Low risk | Low risk | Low risk | Low risk |
| N. Nimbarte, H. Khan, M. D. Sendre, K. Ramteke and S. Wairagade | New Born Baby Cry Analysis and Classification | Low risk | Low risk | Low risk | Low risk | Low risk | Low risk |
| Arsenii Gorin, Cem Subakan, Sajjad Abdoli, Junhao Wang, Samantha Latremouille, Charles Onu | Self-supervised learning for infant cry analysis | Low risk | Low risk | Low risk | Some concerns | Some concerns | Some concerns |
